# Micronutrients in relation to cardiometabolic risk factors among middle-aged and older Chinese adults: protocol for an exploratory longitudinal panel study with multi-omics profiling

**DOI:** 10.3389/fpubh.2026.1871596

**Published:** 2026-07-07

**Authors:** Zhiyue Xu, Xueying Zhao, Lugao Tian, Xinghuang Liu, Min Xiang, Pengcheng Yang, Rongchuan Huang, Xiaotian Xie, Dongke Wang, Ziqian Zhang, Jing He, Chujie Tu, Enli Zhang, Guoxing Zhu, Chao Gu, Hong Yang, Xiaohua Hou, Liangle Yang, Tao Bai

**Affiliations:** 1 Division of Gastroenterology, Union Hospital, Tongji Medical College, Huazhong University of Science and Technology, Wuhan, Hubei, China; 2 Division of Gastroenterology, Central Hospital of Hefeng County, Enshi, Hubei, China; 3 Department of Gastroenterology, Yichang Central People’s Hospital, Yichang, Hubei, China; 4 Key Laboratory of Environment and Health, Ministry of Education, and State Key Laboratory of Environmental Health (Incubating), Department of Occupational and Environmental Health, School of Public Health, Tongji Medical College, Huazhong University of Science and Technology, Wuhan, Hubei, China

**Keywords:** aging population, biomarkers, cardiometabolic risk factors, micronutrients, multi-omics, panel study

## Abstract

The escalating prevalence of cardiometabolic risk factors (CRFs) contributes to increased risks of cardiometabolic diseases. Micronutrients, essential for nearly all physiological functions, have been widely linked to CRFs. However, the joint associations of micronutrient mixtures with CRFs, as well as the potential biological pathways remain incompletely understood. To address this gap, this protocol describes the rationale and design of a longitudinal panel study, which included 205 adults with four repeated seasonal visits in Hefeng County, Enshi Prefecture, Hubei province, China. This project aimed to (1) evaluate the individual and joint associations of biomarker-based micronutrient profiles with CRFs; (2) characterize biological samples using multi-omics approaches to explore candidate biomarkers and potential pathways linking micronutrient profiles with cardiometabolic risk. The planned analyses are expected to help identify relationship of micronutrient profiles with altered biological effects and cardiometabolic diseases risk, and provide a framework for future validation studies and precision nutrition research.

## 1 Introduction

Cardiometabolic diseases, including cardiovascular diseases and diabetes, are leading contributors to morbidity and mortality among middle-aged and older adults (1, 2). Cardiometabolic diseases are driven by a constellation of interconnected cardiometabolic risk factors (CRFs), mainly including obesity, hypertension, dyslipidemia, and hyperglycemia (3). These risk factors not only increase individual susceptibility to adverse cardiovascular events but also impose a significant economic and societal burden on healthcare systems worldwide (4). Therefore, early identification and prevention of CRFs are critical for the control and management of cardiometabolic diseases epidemic, particularly among middle-aged and older populations (5, 6).

Micronutrients, including vitamins and minerals, are essential nutrients required in small quantities to support nearly all physiological functions (7–9). Numerous studies have reported associations between micronutrient and CRFs, but with mixed findings. A meta-analysis showed that higher levels of vitamin D were associated with a significant improvement in lipid profiles and reduction in systolic blood pressure, which were hallmarks of cardiovascular disease (10). Similarly, magnesium and zinc intake has been inversely associated with hyperglycemia, dyslipidemia, hypertension, and markers of inflammation, partly because of their potent antioxidant and anti-inflammatory properties (11, 12). Whereas high iron has been related to increased risks of insulin resistance and type 2 diabetes via introducing oxidative stress and endothelial dysfunction (13, 14). Moreover, for certain micronutrients, such as molybdenum and copper, their associations with CRFs are still largely unclear (15). In addition, previous studies have been constrained by neglecting the joint effects or interactions between different micronutrients on CRFs, inadequate adjustment for important confounders (e.g., chronic diseases or symptoms, dietary habits, and living or working environments), and/or crude exposure assessment that did not account for temporal variability in biomarker-based micronutrient profiles.

Recent advances in high-throughput omics technologies have enabled comprehensive characterization of molecular alterations across multiple biological layers, including gene expression, epigenetic modifications, proteins, gut microbial communities, and metabolites (16, 17). Among these approaches, metabolomics is particularly valuable because metabolites represent downstream products of both endogenous physiological processes and external nutritional and environmental exposures, thereby providing a sensitive snapshot of an individual’s metabolic status (18, 19). Integrating metabolomics with other omics data, such as microbiome, transcriptomic, proteomic, and epigenomic profiles, as well as incorporating network analysis and machine learning, may help identify biomarkers for various CRFs and generate hypotheses regarding pathways through which micronutrient profiles may be associated with cardiometabolic health (20–22). Therefore, this study incorporates untargeted metabolomic profiling of serum, urine, and stool samples and establishes a framework for future multi-omics integration to identify candidate molecular signatures and generate hypotheses regarding potential biological mechanisms underlying observed associations.

To address these knowledge gaps, this exploratory panel study was designed to investigate the individual and joint associations of biomarker-based micronutrient profiles with CRFs among middle-aged and older adults. The study also establishes a multi-omics framework to explore candidate biomarkers and generate hypotheses regarding potential biological pathways linking micronutrient profiles with cardiometabolic risk.

## 2 Material and methods

### 2.1 Study design

A panel study design is ideal for examining the health effects of different exposure time windows, which accounts for autocorrelation in time of within-subject measurements (23–25).

**Figure 1:**
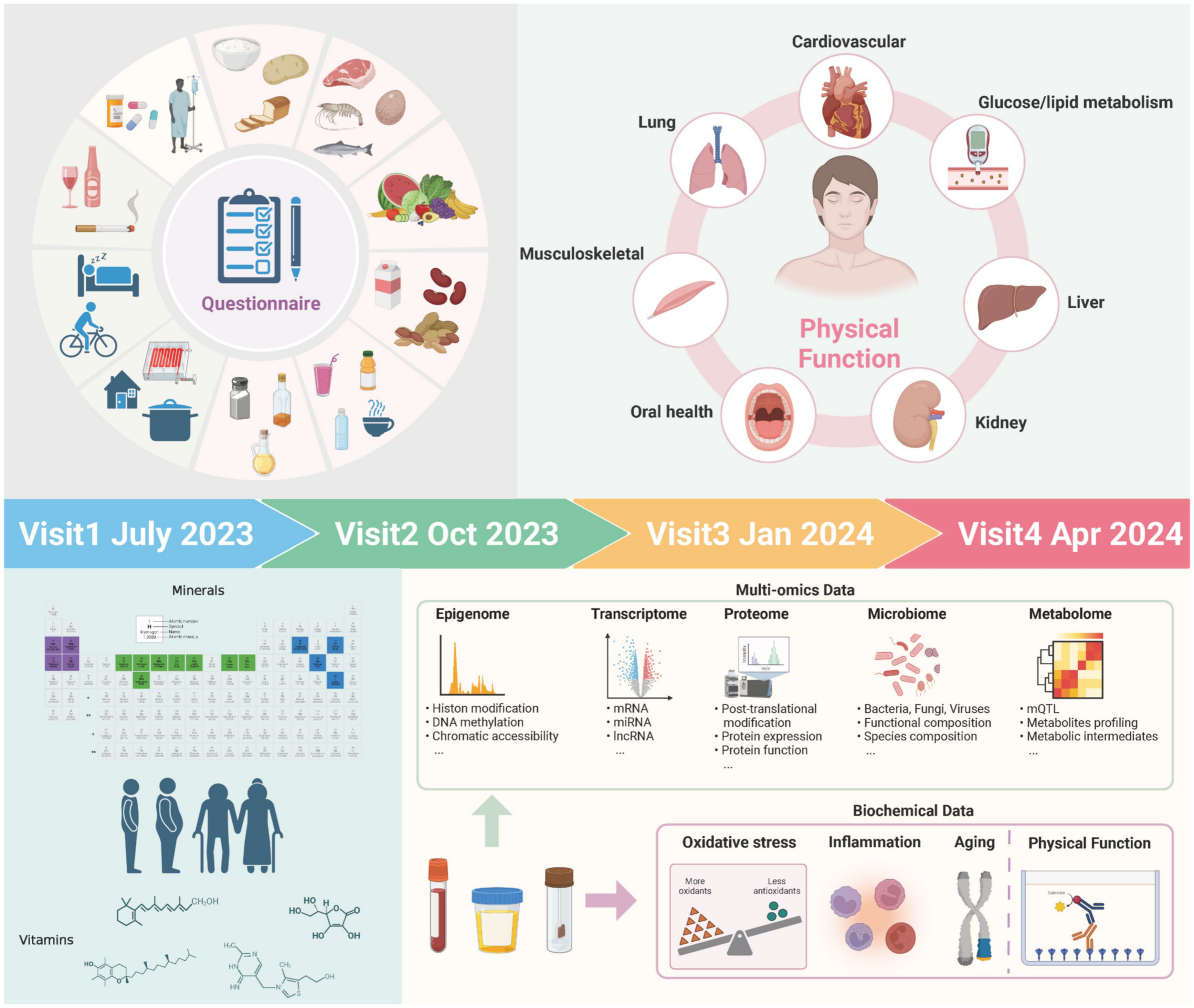
Conceptual diagram of the study design. Created with BioRender.com.

In this study, we implemented a longitudinal panel design to (1) investigate repeated measurements of biomarker-based micronutrient profiles, both as individual exposures and as mixtures, in relation to CRFs; and (2) identify candidate biomarkers associated with micronutrients profiles and the potential mediating roles of them on micronutrient mixtures and CRFs (Figure 1). The research was conducted in Hefeng County, Enshi City, Hubei, China, a region naturally high in selenium (Se) and rich in minerals such as molybdenum (Mo), vanadium (V), nickel (Ni), and zinc (Zn). This unique environmental setting provides an opportunity to explore biomarker-based micronutrient profiles in relation to CRFs (26).

Participants were invited to participate in the 4 repeated visits during the summer (July 29, 2023 to August 6, 2023), autumn (October 28, 2023 to November 4, 2023), winter (January 20, 2024 to January 27, 2024), and spring (April 20, 2024 to April 27, 2024) seasons. The study protocol was reviewed and approved by the Ethics Committee of Union hospital, Tongji Medical College, Huazhong University of Science and Technology (approval number 2023–0525). All participants provided written informed consent prior to their inclusion in the study. The study research was performed in accordance with the Declaration of Helsinki and other relevant guidelines and regulations.

The study illustrates the design for exploring associations between biomarker-based micronutrient profiles and cardiometabolic risk factors among middle-aged and older adults using multi-omics approaches.

### 2.2 Sample size

The sample size was estimated using G*Power V3.1 software. Because this study was designed as an exploratory longitudinal panel study with repeated measurements across four seasonal visits, the sample-size calculation was primarily based on repeated-measures analyses of conventional cardiometabolic risk factors. Assuming an effect size *f* = 0.25 (medium effect), *α* = 0.05, a correlation of 0.5 among repeated measurements, and a dropout rate of 20%, the minimum required sample size (*n* = 180) was calculated to reveal a power of 0.9 in the repeated-measures analyses of variance.

### 2.3 Participants recruitment

Compared to younger individuals, middle-aged and older adults are at higher risk for cardiometabolic diseases (1). Moreover, due to varying physiological needs, dietary habits, and prolonged exposure to micronutrient deficiency or imbalances, this population may be more vulnerable to the detrimental effects of abnormal micronutrient levels.

Participants were recruited using a community-based strategy with assistance from local healthcare providers and community staff in Hefeng County. Potentially eligible residents aged 40–75 years were informed about the study through community announcements and direct contact. Trained investigators explained the study objectives, repeated seasonal visits, questionnaire surveys, health measurements, and biospecimen collection procedures. Eligibility was assessed according to predefined inclusion and exclusion criteria before written informed consent was obtained.

The inclusion criteria were as follows: (1) voluntary participation with signed informed consent; (2) aged 40–75 years; (3) residency at the current address for at least 2 years with no travel plans between July 2023 and April 2024; (4) willingness to comply with study guidelines and maintain regular lifestyle and dietary habits. Exclusion criteria included: (1) language or cognitive barriers; (2) previous diagnosis of diabetes mellitus, coronary heart disease, stroke, or cancer; (3) recent fever or infection within the past month; and (4) recent use of antibiotics, probiotics, or synbiotics.

**Figure 2:**
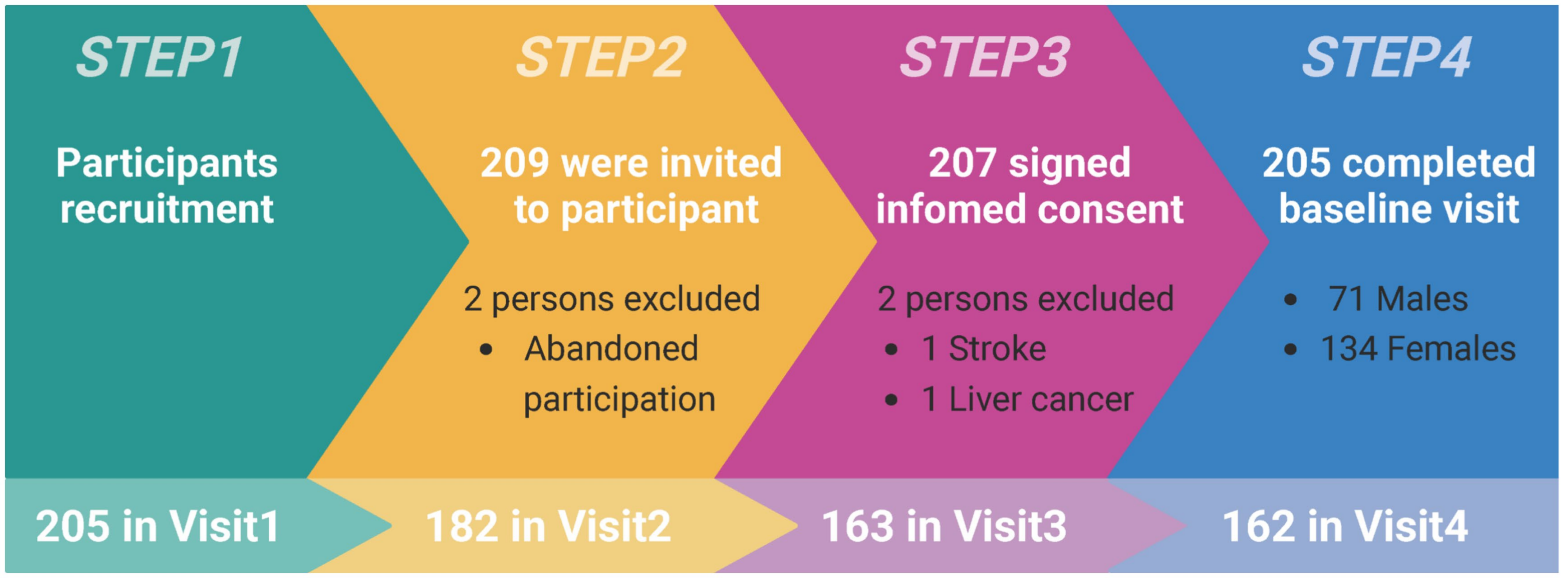
The flow chart of participant enrollment and number of participants in each visit. Created with BioRender.com.

Among 209 invited residents, 2 declined participation, and 207 signed informed consent. Two participants were excluded after screening because of a previous history of stroke or liver cancer; therefore, 205 participants completed the baseline visit. Among those participants, 182 completed the second visit, 163 completed the third visit, 162 completed the fourth visit, resulting in a total of 712 person-visits (Figure 2). Reasons for missed visits were not systematically recorded for each participant. Based on field communication, nonparticipation in follow-up visits was mainly related to temporary personal affairs, work-schedule conflicts, or reduced willingness to continue because some participants considered themselves healthy and did not perceive a need for repeated examinations. To evaluate potential attrition bias in subsequent analyses, baseline demographic characteristics, lifestyle factors, and major cardiometabolic indicators will be compared between participants who completed all four visits and those with incomplete follow-up. Each participant was assigned a unique ID number for study purposes.

### 2.4 Questionnaires and health measurements

Four visits were conducted from July 2023 to April 2024, each lasting 8–9 days and covering both weekdays and weekends. During each visit, 20–30 participants were scheduled daily from 8 a.m. to 12 p.m. to complete questionnaires, undergo health assessments, and provide biological samples. The timing of these visits, corresponding to the distinct local seasons—summer, autumn, winter, and spring—was advantageous for monitoring seasonal variations in lifestyle and dietary habits.

All questionnaires were administered by trained investigators through face-to-face interviews. The questionnaire covered the following areas: (1) personal information (age, gender, nation, marriage, education level); (2) occupational and employment history; (3) medical history, family medical history and medication history; (4) lifestyle factors (smoking status, alcohol consumption, physical activity and sleep patterns); (5) household characteristics (household composition, residential characteristics, annual income, type of house, ventilation, use of heater, cooking frequency and etc.); (6) oral health issues; (7) gastrointestinal symptoms in past 1 month; (8) dietary intake in preceding 4 weeks.

Dietary intake was assessed using a semi-quantitative food frequency questionnaire (FFQ) administered by trained investigators through face-to-face, one-on-one interviews. The FFQ used in this study was adapted from a semi-quantitative FFQ previously developed for pregnant women in Wuhan, China, by the research team that included some members of the present project team. The original FFQ has been reported to have good reproducibility and relative validity (27).

The questionnaire covered 13 major food categories and commonly consumed food items, including cereals and grains, livestock and poultry meat, aquatic products, eggs, beans and bean products, vegetables, fruits, nuts, milk and dairy products, beverages, oils and fats, processed foods, and condiments. For major food items, participants were asked to report both consumption frequency and average amount consumed per eating occasion. Consumption frequency referred to the number of times a specific food was consumed during the preceding month, ranging from “consumed only once in the past 4 weeks” to “consumed five or more times per day.”

To improve the accuracy and consistency of portion-size estimation, all dietary investigators received standardized training before the survey. During the interview, standard bowls, plates and commonly used tableware were used to help participants estimate intake amounts. The questionnaire also included edible-portion or conversion factors for selected foods to further standardize intake estimation. Total energy intake will be estimated by combining reported consumption frequency, average intake amount per eating occasion, and energy values from the Chinese Food Composition Database.

During each visit, the following health measurements were conducted: (1) height, weight, blood pressure, waist circumference, hip circumference, grip strength and body fat; (2) lung function indices, fractional exhaled nitric oxide (FeNO) and fractional exhaled carbon monoxide (FeCO); (3) 12-lead electrocardiogram (ECG) and 10-min heart rate variability (HRV); (4) arterial stiffness, bone mineral density (BMD); (5) ultrasound (heart, carotid artery, vertebral artery, urinary system, liver, gallbladder, pancreas, spleen), Transcranial doppler (TCD) and liver elastography; (6) routine blood examination, routine urine examination, routine stool examination, fecal occult blood test (FOBT), liver function, renal function, electrolytes, coagulation function, fasting blood glucose (FBG), glycated hemoglobin (HbA1c) and blood lipids. Besides, methylation screening for colorectal cancer and C14 urea breath test were added in third and fourth visit, respectively. The ultrasound, laboratory tests, ECG and C14 urea breath test were conducted by clinical laboratory of Central Hospital of Hefeng County, and the other items were carried out by trained personnel using professional instruments.

### 2.5 Biological samples collection

During each visit, biological samples, including fasting venous blood, urine, and stool, were collected from participants according to standardized operating procedures. Participants were instructed to fast from 10:00 p.m. on the evening before sample collection, with only water permitted if necessary. Venous blood samples were collected between 8:00 a.m. and 10:00 a.m. the following morning using both gel-separating tubes and EDTA-coated tubes.

For serum preparation, blood samples collected in gel-separating tubes were allowed to clot upright at room temperature for 30 min and then centrifuged at 1,500 × g for 10 min at 4 °C. For plasma and blood-cell preparation, EDTA-anticoagulated blood samples were gently inverted several times immediately after collection and centrifuged under the same conditions. After centrifugation, serum, plasma, blood cells, and blood clots were carefully separated and aliquoted into sterile, nuclease-free cryovials. Liquid samples were transferred using calibrated pipettes with sterile, nuclease-free filtered tips to minimize contamination and sample loss.

Morning urine samples and stool samples were collected using individually packaged sterile disposable containers. Immediately after collection, urine and stool samples were placed in pre-chilled insulated transport containers containing frozen ice packs and transported to the sample-processing room as soon as possible. During temporary storage, transportation, and aliquoting, samples were maintained under low-temperature conditions. Urine samples were mixed gently before aliquoting. Stool samples were homogenized as appropriate and aliquoted using individually packaged sterile, nuclease-free disposable spoons. All aliquoting procedures were performed using sterile, nuclease-free consumables, and each biospecimen was divided into multiple aliquots to avoid repeated freeze–thaw cycles in subsequent analyses.

After aliquoting, all cryovials were immediately transferred to a biobank and stored at −80 °C to preserve long-term sample integrity. Each sample tube was labeled with the collection time, unique participant ID, biospecimen type, visit number, and aliquot number to ensure accurate tracking and identification throughout the study.

### 2.6 Micronutrients measurements

Micronutrients consist of vitamins and minerals that are crucial for the body’s proper functioning, yet required only in small quantities. Vitamins include fat-soluble types (A, D2, D3, E, K1) and water-soluble types (C, B1, B2, B3, B5, B6, B7, B9, B12), while minerals are divided into major essential minerals (Ca, K, Na, Cl, Mg, P), essential trace elements (Fe, Zn, Cu, Mn, Se, Co, Mo, Cr, I) and selected nutrition-related and environmentally relevant trace elements (Ni, V).

Vitamins were accurately measured by using high-performance liquid chromatography (HPLC) and liquid chromatography–tandem mass spectrometry (LC–MS/MS) (28, 29). For minerals, techniques including atomic absorption spectroscopy and inductively coupled plasma mass spectrometry (ICP-MS) were utilized to determine the accurate concentration in biological samples (30).

### 2.7 CRFs and biomarkers

CRFs refer to a group of interrelated biological and behavioral determinants that collectively increase the likelihood of developing cardiovascular diseases and type 2 diabetes (3). Conventional CRFs, including obesity, hypertension, dyslipidemia, and hyperglycemia, were well-recognized due to their contribution to systemic vascular and metabolic dysfunctions (3, 31). Moreover, emerging elements such as chronic inflammation, oxidative stress, and gut microbiota dysbiosis have expanded the scope of these risk factors, underscoring their multifactorial nature (32, 33).

To improve the focus of the study and align the outcome definition with the sample-size justification and statistical analysis plan, we organized the measured indicators into a hierarchical outcome framework. The primary outcome domain comprises conventional CRFs, including adiposity-related indices, blood pressure, glucose metabolism, and lipid metabolism. Secondary outcomes include functional and biochemical biomarkers related to cardiovascular function, other organ or tissue functions, oxidative stress, inflammation, and aging. Omics-derived molecular features will be considered exploratory outcomes or candidate mediators for biomarker discovery, pathway enrichment, and hypothesis generation. The major outcome domains are summarized in Table 1, and the full list of measured indicators is provided in Supplementary Table 1.

(1) Primary outcomes: conventional CRFs.

**Table 1:** Hierarchical outcome framework and representative indicators.

Outcome level	Domain	Representative indicators or measurements
Primary outcomes	Conventional cardiometabolic risk factors	Adiposity-related indices: body mass index, waist circumference, hip circumference, waist-to-hip ratio, and body fat; Blood pressure; Glucose metabolism indicators: fasting blood glucose, glycated hemoglobin, glycosylated serum protein, insulin, and C-peptide; Lipid metabolism indicators: total cholesterol, triglycerides, low-density lipoprotein cholesterol, high-density lipoprotein cholesterol, apolipoprotein A, apolipoprotein B, and lipoprotein (a).
Secondary outcomes	Expanded cardiovascular function	Heart rate; electrocardiogram parameters; heart rate variability; echocardiography; carotid and vertebral artery ultrasound; transcranial Doppler; arterial stiffness indicators, including ankle-brachial index and pulse wave velocity.
	Functions of other organs/tissues	General health status; pulmonary function and airway inflammation; hepatic and renal function; musculoskeletal function; oral health; coagulation function; routine blood, urine, and stool examination parameters.
Secondary biomarkers	Oxidative stress, inflammation, and aging	Oxidative-stress biomarkers: 8-hydroxydeoxyguanosine, 8-isoprostane, superoxide dismutase, glutathione, glutathione peroxidase, and malondialdehyde; Inflammatory markers: interleukins, tumor necrosis factor-α, interferon-γ, hypersensitive C-reactive protein, and related cytokines; Aging-related biomarkers: telomere length, mitochondrial DNA copy number, and selected aging-related metabolites.
Exploratory outcomes and candidate mediators	Multi-omics-derived molecular features	Metabolomics, microbiomics, epigenomics, transcriptomics, proteomics, and other available omics-derived features for exploratory biomarker discovery, pathway enrichment, and mediation analyses.

The full list of measured indicators and potential biomarkers is provided in Supplementary Table 1.

Micronutrient profiles have been associated with cardiovascular function and lipid-glucose metabolism (34, 35), which are closely related to the development of cardiovascular and metabolic diseases in middle-aged and older populations. In this study, conventional CRFs were defined as the primary outcomes because they are clinically established indicators of cardiometabolic risk and are directly aligned with the sample-size justification and primary repeated-measure analyses. These primary outcomes include adiposity-related indices, blood pressure, glucose metabolism, and lipid metabolism.

Adiposity is a central component of cardiometabolic risk, as excess body weight and abnormal fat distribution are closely related to insulin resistance, hypertension, dyslipidemia, and systemic inflammation. Blood pressure is another core indicator of cardiovascular risk and reflects both vascular function and metabolic burden. Glucose metabolism indicators are essential for characterizing hyperglycemia, insulin secretion, and early metabolic dysregulation, whereas lipid metabolism indicators reflect atherogenic risk and metabolic homeostasis. Therefore, these conventional CRFs provide the main outcome framework for evaluating the relationship between biomarker-based micronutrient profiles and cardiometabolic health.

(2) Secondary outcomes: expanded cardiovascular function and functions of other organs/tissues.

In addition to conventional CRFs, expanded cardiovascular functional measures and functions of other organs/tissues were assessed as secondary outcomes to provide a broader characterization of cardiometabolic health. Beyond routine parameters such as heart rate and electrocardiogram indicators, health assessments included heart rate variability (HRV), echocardiography, carotid and vertebral artery ultrasound, transcranial Doppler (TCD), and arterial stiffness. These measurements may capture subclinical cardiovascular functional changes that are not fully reflected by conventional CRFs alone.

The decline in physical function and organ function is a gradual process associated with aging, and abnormal micronutrient levels have been linked to dysfunctions in multiple organ systems (36, 37). Besides, there is a close link between different organs and tissues, which constructed an interactive network of CRFs (33). Therefore, we conducted comprehensive assessments of various organ functions, including general health, pulmonary function, hepatic and renal function, and musculoskeletal function.

(3) Secondary biomarkers: oxidative stress, inflammation and aging.

Oxidative stress and inflammation are linked to pathophysiological processes that play a crucial role in aging and age-related diseases, especially for cardiovascular and metabolic diseases (38–41). Understanding how micronutrient profiles are associated with these pathways may provide insight into potential mechanisms linking nutritional status with age-related cardiometabolic risk.

Accordingly, oxidative-stress indicators such as 8-hydroxydeoxyguanosine (8-OHdG), as well as inflammatory markers such as interleukins will be measured as secondary biochemical biomarkers. These biomarkers may help characterize biological perturbations that accompany changes in conventional CRFs.

Telomere length and mitochondrial DNA copy numbers (mtDNA-CN) are typical biomarkers of aging (42), which may offer additional insights into how micronutrient imbalance is associated with cardiometabolic disease (41, 43, 44). Cellular metabolism, which is deeply intertwined with aging, involves several metabolites strongly associated with age and geroprotective effects (45). Candidates for future testing include NAD+, alpha-ketoglutarate, tryptophan, methionine, and spermidine (42).

(3) Exploratory outcomes and candidate mediators based on multi-omics approaches.

Advances in multi-omics technologies may help identify novel biomarkers for cardiometabolic risk, and provide insights into molecular pathways underlying metabolic health and disease (46). In this study, untargeted metabolomics will be performed on serum, urine, and stool samples to capture systemic, urinary, and gut-related metabolic features. Candidate metabolites identified from untargeted profiling may be further examined using targeted assays for validation where applicable. By integrating data from the epigenome, transcriptome, microbiome, metabolome and proteome, these methods may provide a comprehensive view of the interplay between biological systems.

Epigenetic modifications, influenced by dietary and environmental factors, may reveal early molecular signatures of cardiometabolic dysfunction (47). Meanwhile, minerals such as selenium and nutrients like vitamin D have been shown to regulate gene expression and translation through epigenetic pathways, including DNA methylation, post-translational histone modifications, and chromatin structure (48–50). Concurrently, growing evidence has highlighted the role of gut microbiota and microbiota-derived compounds in host metabolism and the pathogenesis of metabolic diseases (51). In addition, numerous endogenous small molecule metabolites have also served as potential biomarkers for pathophysiological changes, and diseases identification (19, 52).

Taken together, omics-derived molecular features will be treated as exploratory outcomes or candidate mediators in this protocol. These analyses are intended for biomarker discovery, pathway enrichment, and hypothesis generation regarding potential biological mechanisms linking micronutrient profiles with CRFs.

### 2.8 Multi-omics quality control and reproducibility

For metabolomics and other omics assays, samples will be randomized across analytical batches. Pooled QC samples generated by mixing aliquots from study samples, procedural blanks, and internal standards will be included to monitor analytical stability and contamination. Repeated freeze–thaw cycles will be avoided. Features with high missingness or poor reproducibility in QC samples will be excluded according to prespecified thresholds, such as missingness exceeding 20–30% or QC coefficient of variation > 30%, depending on the omics platform. Batch effects will be evaluated and corrected using appropriate methods, such as QC-based signal correction or ComBat where applicable. Multiple testing will be controlled using the Benjamini-Hochberg false discovery rate. Candidate biomarkers identified from discovery analyses will be prioritized for validation using targeted assays or cross-platform confirmation where feasible.

### 2.9 Ethics and data management

This study protocol was reviewed and approved by the Ethics Committee of Union Hospital, Tongji Medical College, Huazhong University of Science and Technology (approval number 2023–0525). All participants provided written informed consent before enrollment. The informed-consent process covered repeated questionnaire surveys, health assessments, biospecimen collection, long-term biospecimen storage, and future secondary analyses related to the approved research scope.

To protect participant privacy, each participant was assigned a unique study identification number. Biospecimens and research data were labeled and managed using study IDs rather than directly identifiable personal information. Personally identifiable information was stored separately from analytical datasets and was accessible only to authorized study personnel. Electronic data were stored in password-protected databases with restricted access, and paper documents were kept in secure locations. Data checking, cleaning, and analysis were performed using de-identified datasets.

Because this study involves blood, urine, stool, biobanking, microbiome profiling, metabolomics, and other potential omics analyses, additional measures were implemented to protect sensitive biological and omics-related information. Access to biospecimens and omics datasets will be restricted to authorized investigators and used only for ethically approved research purposes. Biospecimens will be stored in the study biobank at −80 °C according to institutional regulations and the approved consent scope. Any future secondary use beyond the current approved research scope will require additional ethical approval where applicable.

Clinically relevant abnormal findings identified during routine health assessments or laboratory examinations will be communicated to participants, who will be advised to seek further medical evaluation when necessary. De-identified data and analysis code may be made available from the corresponding author upon reasonable request, subject to ethical approval, participant privacy protection, and institutional data-sharing policies.

### 2.10 Statistical analysis

Descriptive statistics will be used to summarize demographic characteristics, lifestyle factors, dietary variables, biomarker-based micronutrient profiles, and cardiometabolic risk factors. Continuous variables will be presented as means ± standard deviations or medians with interquartile ranges, depending on their distributions. Categorical variables will be summarized as numbers and percentages.

For single-exposure analyses, linear mixed-effects (LME) models will be used to evaluate associations between individual biomarker-based micronutrient levels and continuous cardiometabolic outcomes. The primary LME models will include a participant-specific random intercept to account for within-subject correlations across repeated measurements over time. Fixed effects will include potential confounders selected based on prior evidence, clinical relevance, and directed acyclic graph considerations, such as age, sex, ethnicity, education, smoking status, alcohol consumption, physical activity, total energy intake, season, medication use, and outcome-specific baseline health conditions. Random slopes for visit or time will not be included in the primary models because the limited number of repeated visits may reduce model stability and lead to convergence problems. However, models including random slopes will be evaluated in sensitivity analyses when convergence and model-fit indices support their use. For binary cardiometabolic outcomes, such as hypertension, dyslipidemia, or impaired glucose metabolism, mixed-effects logistic regression models will be used.

For mixture analyses, weighted quantile sum regression (WQS regression) will first be used to evaluate the overall joint association of micronutrient mixtures with cardiometabolic risk factors and to identify components that contribute most strongly to the mixture association. Because associations between micronutrients and outcomes may be non-linear, and because complex interactions may exist among mixture components, Bayesian kernel machine regression (BKMR) will also be applied as an exploratory approach to assess potential non-linear exposure-response relationships and high-dimensional interactions. For pairwise interactions with biological plausibility or *a priori* hypotheses, product terms will be included in mixed-effects models to evaluate interactions on the multiplicative scale. For binary cardiometabolic outcomes, such as hypertension, dyslipidemia, or impaired glucose metabolism, additive interactions will be further assessed using the relative excess risk due to interaction (RERI), attributable proportion due to interaction (AP), and synergy index (S) when sample size and model stability permit.

For multi-omics analyses, raw data will be preprocessed using platform-specific pipelines, including quality filtering, normalization, transformation, scaling, and batch-effect assessment or correction where appropriate. Omics features with excessive missingness or poor reproducibility in quality-control samples will be excluded according to prespecified criteria. Exploratory feature discovery will be performed using mixed-effects models or other appropriate methods that account for repeated measurements. Dimension-reduction or classification approaches, such as orthogonal partial least-squares-discriminant analysis (OPLS-DA), may be used as supplementary exploratory tools rather than as the primary basis for statistical inference. For omics features associated with biomarker-based micronutrient profiles or cardiometabolic risk factors, pathway enrichment analyses will be conducted to identify potentially perturbed biological pathways.

Mediation analyses will be conducted only when a plausible temporal and biological exposure-mediator-outcome sequence exists. Candidate mediators may include selected metabolites, microbiome features, inflammatory markers, oxidative-stress biomarkers, or other omics-derived features. These analyses will be considered exploratory because of the observational design and the complexity of repeated-measure data. The direct and indirect associations between micronutrient profiles and cardiometabolic risk factors through candidate biomarkers will be estimated using appropriate mediation models, with cautious interpretation regarding causality.

For analyses involving multiple exposures, outcomes, or high-dimensional omics features, multiple testing will be controlled using the Benjamini-Hochberg false discovery rate (FDR) where appropriate. Model stability will be assessed using internal validation procedures, such as repeated training-validation splits, bootstrapping, or sensitivity analyses with alternative model specifications, depending on the analytical method. Missing data and unbalanced repeated measurements will be handled primarily within mixed-effects models under the missing-at-random (MAR) assumption. The primary analyses will include all available person-visits rather than restricting analyses to participants with complete data from all four visits. For subsequent analytical manuscripts based on specific research questions, the proportion and pattern of missing data will be summarized for major exposures, outcomes, and covariates. Sensitivity analyses may include complete-case analyses, alternative covariate adjustment sets, exclusion of influential observations, multiple imputation for missing covariates, or inverse probability weighting to evaluate the influence of missing data and attrition, depending on the missing-data pattern, sample size, and model stability. Given the exploratory nature of the study, mixture modeling, non-linear analyses, interaction analyses, multi-omics discovery, and mediation analyses will be interpreted as hypothesis-generating rather than confirmatory.

## 3 Discussion

Cardiometabolic diseases, including cardiovascular diseases and diabetes, remain major contributors to morbidity and mortality worldwide. These conditions arise from complex interactions among environmental exposures, lifestyle factors, aging-related physiological changes, and genetic susceptibility. Micronutrients participate in diverse physiological processes, including oxidative stress regulation, inflammatory response modulation, and metabolic homeostasis. Imbalances in micronutrient status may therefore be associated with cardiometabolic risk, particularly among middle-aged and older adults. Recent advances in multi-omics technologies provide opportunities to explore candidate biomarkers and potential biological pathways linking biomarker-based micronutrient profiles with cardiometabolic health.

This study protocol describes a longitudinal platform for assessing biomarker-based micronutrient profiles, conventional CRFs, secondary functional and biochemical biomarkers, and exploratory multi-omics features across four seasonal visits. The repeated-measure design may help characterize temporal variations in micronutrient profiles and their associations with cardiometabolic risk indicators. However, because this is an observational protocol, the planned analyses will be interpreted as exploratory and hypothesis-generating, and causal inference will remain limited. The planned integration of multi-omics data with environmental and lifestyle factors, such as physical activity, dietary quality, and socioeconomic status, may support a broader exploration of the determinants of cardiometabolic risk among middle-aged and older adults.

Future studies should further evaluate less-studied micronutrients, such as vanadium and molybdenum, and validate candidate biomarkers or pathways identified from exploratory analyses. Larger independent cohorts and, where appropriate, intervention studies will be needed to confirm whether micronutrient-related strategies can contribute to cardiometabolic disease prevention.

The main strengths of this exploratory panel study include its longitudinal design with short follow-up intervals, repeated measurements of exposures and outcomes, and biospecimen collection across four seasonal visits, which may improve the characterization of temporal variability in micronutrient profiles and cardiometabolic indicators. However, several limitations should also be noted. First, this study could not establish causality between exposure and outcomes. Second, the generalizability of the study should be interpreted with caution. The study population consists of community-dwelling middle-aged and older adults from Hefeng County. The study site was selected according to the research objective because of its distinctive mineral geochemical background, which may limit direct extrapolation to populations living in regions with different environmental, dietary, and trace-element exposure patterns. In addition, community-based volunteer recruitment and attrition during repeated visits may introduce selection bias. Therefore, the findings should not be interpreted as nationally representative estimates for all middle-aged and older adults or all populations at cardiometabolic risk. Third, although biomarker-based micronutrient profiles were used as the primary exposure indicators in this study, the influence of other unmeasured environmental chemicals or co-exposures with similar sources cannot be fully excluded, which may lead to residual confounding.
