# Supplementary material for: Micronutrients in relation to cardiometabolic risk factors among middle-aged and older Chinese adults: protocol for an exploratory longitudinal panel study with multi-omics profiling
Source: Front Public Health. 2026 Jul 7;14:1871596. doi: 10.3389/fpubh.2026.1871596 (PMC13385261; doi:10.3389/fpubh.2026.1871596)
Supplement: Supplementary file 1 [file Table_1.docx]

Supplementary Table 1. Detailed list of measured indicators and potential biomarkers

| **Effect indicators Category** | **Biological Effects** | **Biomarkers** | **Biosample/ measurement method** | **Reference** |
| --- | --- | --- | --- | --- |
| Cardiovascular function and lipid-glucose metabolism | Cardiovascular function | Blood pressure | Electronic blood pressure monitor | **^1-4^** |
|  |  | Heart rate, P wave, PR interval, QRS complex, QT interval, ST segment, T wave | ECG |  |
|  |  | SDNN, SDNN-index, SDANN, RMSSD, SDSD, PNN50, Low-frequency power (P1), High-frequency power (P2), Ultralow-frequency power (P3), Total power | HRV |  |
|  |  | Peak flow velocity (PFV) and pressure gradient (PG) of tricuspid valve, aortic valve and pulmonary valve; E-wave and A-wave of mitral valve and septal mitral annulus; Ejection fraction (EF); Fractional shortening (FS) | Cardiac ultrasound |  |
|  |  | Atherosclerotic plaque diameter, Internal diameter of vertebral artery | Carotid/vertebral artery ultrasound |  |
|  |  | Depth, systolic peak velocity (Vs), Diastolic peak velocity (Vd), Mean flow velocity (Vm), Pulsatility index (PI), Resistive index (RI), and Systolic/Diastolic ratio (S/D) of basilar artery (BA), left vertebral artery (LVA), right vertebral artery (RVA), right middle cerebral artery (RMCA), right anterior cerebral artery (RACA), right posterior cerebral artery (RPCA), left middle cerebral artery (LMCA), left anterior cerebral artery (LACA), and left posterior cerebral artery (LPCA). | Transcranial doppler (TCD) |  |
|  |  | Ankle-brachial index (ABI), Pulse wave velocity (PWV) | Arteriosclerosis detector |  |
|  |  | Homocysteine, B-type natriuretic peptide (BNP), Fibrinopeptide A (FPA), CD14, Granin-like neuroendocrine peptide precursor (ProSAAS), Membrane-associated progesterone receptor component 1 (PGRMC1), Sodium/potassium-transporting ATPase gamma chain (FXYD2), Fibrinogen-alpha chain (FGA), alpha1 anti-trypsin (AAT), Fibulin-1 | Serum, urine |  |
|  | Glucose/lipid metabolism function | Waist circumference, Hip circumference, Body fat | Physical examination | **^5,6^** |
|  |  | Fasting blood glucose (FBG), Glycated hemoglobin (HbA1c), Glycosylated serum protein (GSP), Insulin, C-peptide; Apolipoprotein A (APOA), Apolipoprotein B (APOB), Total cholesterol (TC), Triglyceride (TG), Low density lipoprotein cholesterol (LDL-CH), High density lipoprotein cholesterol (HDL-CH), Lipoprotein (a) (Lp (a)) | Serum |  |

Supplementary Table 1 (continued)

| Functions of  other organs/tissues | General condition | Height, Weight |  |  |
| --- | --- | --- | --- | --- |
|  | Lung function and  airway inflammation | VC, FVC, FEV1, FEV3, MMF, PEF, FEF25, FeNO50, FeNO200, CaNO, eCO |  | **^7^** |
|  | Liver function | Liver Stiffness, Liver fat attenuation coefficient, Osteopontin (OPN), Mesencephalic astrocyte-derived neurotrophic factor (MANF), Alanine aminotransferase (ALT), Aspartate Aminotransferase (AST), Total protein (TP), Albumin (ALB), Globulin (GLO), Total bilirubin (TBIL), Direct bilirubin (DBIL), Indirect bilirubin (IDBIL), Alkaline phosphatase (ALP), Gamma-glutamyl transferase (GGT), Total bile acids (TBA) | liver elastography, serum | **^8^** |
|  | Renal function | Blood urea nitrogen (BUN), Creatinine (CREA), uric acid (UA), Cystatin C (Cys-C), Carbon dioxide combining power (CO2CP), estimated glomerular filtration rate (eGFR) | Serum | ^9^ |
|  | Skeletal/muscle function | Speed of sound (SOS), T-score, Z-score, grip strength | Bone sonometer  Electronic hand dynamometer | ^10^ |
|  | Oral health | Number of teeth lost, Age at first tooth loss, Number of dentures, Frequency of oral discomfort, Dietary restrictions due to oral problems | Physical examination, Questionnaire | ^11^ |
|  | Coagulation function | Prothrombin time (PT), Prothrombin time international normalized ratio (PT-INR), Activated partial thromboplastin time (APTT), Fibrinogen (FIB), Thrombin time (TT) | Plasma |  |
|  | Routine examination | Red blood cell count (RBC), Hemoglobin (HGB), Hematocrit (HCT), Mean corpuscular volume (MCV), Mean corpuscular hemoglobin (MCH), Mean corpuscular hemoglobin concentration (MCHC), Red cell distribution width - coefficient of variation (RDW-CV), Red cell distribution width - standard deviation (RDW-SD);  Platelet count (PLT), Mean platelet volume (MPV), Plateletcrit (PCT), Platelet distribution width (PDW), Platelet-large cell ratio (P-LCR);  White blood cell count (WBC), Neutrophil count (NEUT), Lymphocyte count (LYMPH), Monocyte count (MONO), Eosinophil count (EO), Basophil count (BASO), Neutrophil percentage (NEUT%), Lymphocyte percentage (LYMPH%), Monocyte percentage (MONO%), Eosinophil percentage (EO%), Basophil percentage (BASO%); | Blood (Routine blood examination) |  |

Supplementary Table 1 (continued)

|  |  | Urine protein (PRO), Urine occult blood (BLD), Nitrite (NIT), Urinary white blood cells (U-WBC), Urine glucose (GLU), Urine ketone bodies (KET), Urine bilirubin (BIL), Urine pH, Specific gravity (SG), Urine leukocyte (LEU), Urobilinogen (UBG), | Urine (Routine urine examination) |  |
| --- | --- | --- | --- | --- |
|  |  | Stool consistency, Stool white blood cells, Stool red blood cells, Occult blood | Stool (Routine stool examination) |  |
| Oxidative stress, inflammation and aging | Oxidative stress | 8-hydroxydeoxyguanosine (8-OHdG), 8-isoprostane, Superoxide dismutase (SOD), Glutathione (GSH), Glutathione peroxidase (GSH-Px), Malondialdehyde (MDA) | Urine, Serum | ^1,12-15^ |
|  | Inflammation factors | Interleukin (IL-1β, IL-2, IL-4, IL-5, IL-6, IL-7, IL-8, IL-10, IL-12, IL-13, IL-17, IL-21, IL-23), Tumor necrosis factor-α (TNF-α), Interferon-γ (IFN-γ), Platelet-activating factor (PAF), Hypersensitive C-reactive protein (hs-CRP), Macrophage inflammatory protein-1α (MIP-1α), Macrophage inflammatory protein-1β (MIP-1β), Macrophage inflammatory protein-3α (MIP-3α), Fractalkine (FKN) | Serum |  |
|  | Aging | Telomere length, mitochondrial DNA copy number (mtDNA-CN)  NAD+, alpha-Ketoglutarate, Tryptophan, Methionine, Spermidine | Blood cell, serum |  |
| Omics | Epigenetics | DNA methylation, Histone modifications, lncRNA, miRNA | Blood DNA, Plasma | ^16-20^ |
|  | Transcriptomics | mRNA (e.g. Gene involved in metabolism, inflammation, oxidative stress, aging, nutrient transporters) | Blood |  |
|  | Proteomics | Proteins (e.g. Inflammatory cytokines, Hormones related to metabolism, Enzymes involved in oxidative stress and antioxidant defense) | Serum |  |
|  | Microbiomics | Bacteria, Fungi, Viruses, Archaea | Stool |  |
|  | Metabolomics | Metabolites (e.g. Amino acids, Nucleotides, Fatty acids, Bile acids, TMAO (Trimethylamine N-oxide) and related compounds, Organic acids, Carnitine, Aromatic compounds, Carbohydrates, Indoles, Amines, Vitamins, Glycerophospholipids, Sphingolipids, Triglycerides, Cholesteryl Esters) | Serum, Stool, Urine |  |

**References**

1 Amorim, J. A. *et al.* Mitochondrial and metabolic dysfunction in ageing and age-related diseases. *Nat Rev Endocrinol* **18**, 243-258, doi:10.1038/s41574-021-00626-7 (2022).

2 Zhang, Y. *et al.* Lung function assessment and its association with blood chromium in a chromate exposed population. *Sci Total Environ* **818**, 151741, doi:10.1016/j.scitotenv.2021.151741 (2022).

3 Gillies, N. A. *et al.* Vitamin B and One-Carbon Metabolite Profiles Show Divergent Associations with Cardiometabolic Risk Markers but not Cognitive Function in Older New Zealand Adults: A Secondary Analysis of the REACH Study. *J Nutr* **153**, 3529-3542, doi:10.1016/j.tjnut.2023.10.012 (2023).

4 Szabo, T. M. *et al.* Total 25-Hydroxyvitamin D Is an Independent Marker of Left Ventricular Ejection Fraction in Heart Failure with Reduced and Mildly Reduced Ejection Fraction. *Biomolecules* **13**, 1578, doi:10.3390/biom13111578 (2023).

5 Dandona, P., Aljada, A. & Bandyopadhyay, A. Inflammation: the link between insulin resistance, obesity and diabetes. *Trends Immunol* **25**, 4-7, doi:10.1016/j.it.2003.10.013 (2004).

6 Asprouli, E. *et al.* Evaluation of Plasma Trace Elements in Different Stages of Nonalcoholic Fatty Liver Disease. *Biol Trace Elem Res* **188**, 326-333, doi:10.1007/s12011-018-1432-9 (2019).

7 Lin, P. D. *et al.* Non-essential and essential trace element mixtures and kidney function in early pregnancy - A cross-sectional analysis in project viva. *Environ Res* **216**, 114846, doi:10.1016/j.envres.2022.114846 (2023).

8 Zhong, W. *et al.* Dietary Diversity, Micronutrient Adequacy and Bone Status during Pregnancy: A Study in Urban China from 2019 to 2020. *Nutrients* **14**, 4690, doi:10.3390/nu14214690 (2022).

9 Akinkugbe, A. A. *et al.* Metal mixtures and oral health among children and adolescents in the National Health and Nutrition Examination Survey (NHANES), 2017-2020. *Int J Hyg Environ Health* **257**, 114335, doi:10.1016/j.ijheh.2024.114335 (2024).

10 Mizuno, Y. *et al.* Determinants of oxidative stress among indigenous populations in Northern Laos: Trace element exposures and dietary patterns. *Sci Total Environ* **868**, 161516, doi:10.1016/j.scitotenv.2023.161516 (2023).

11 Valla, F. V. *et al.* Multiple Micronutrient Plasma Level Changes Are Related to Oxidative Stress Intensity in Critically Ill Children. *Pediatr Crit Care Med* **19**, e455-e463, doi:10.1097/PCC.0000000000001626 (2018).

12 Filgueiras, M. S., Rocha, N. P., Novaes, J. F. & Bressan, J. Vitamin D status, oxidative stress, and inflammation in children and adolescents: A systematic review. *Crit Rev Food Sci Nutr* **60**, 660-669, doi:10.1080/10408398.2018.1546671 (2020).

13 Thaler, R. *et al.* Vitamin C epigenetically controls osteogenesis and bone mineralization. *Nat Commun* **13**, 5883, doi:10.1038/s41467-022-32915-8 (2022).

14 Gu, K. *et al.* Iron overload induces colitis by modulating ferroptosis and interfering gut microbiota in mice. *Sci Total Environ* **905**, 167043, doi:10.1016/j.scitotenv.2023.167043 (2023).

15 Mietus-Snyder, M. *et al.* Next Generation, Modifiable Cardiometabolic Biomarkers: Mitochondrial Adaptation and Metabolic Resilience: A Scientific Statement From the American Heart Association. *Circulation* **148**, 1827-1845, doi:10.1161/CIR.0000000000001185 (2023).

16 Yim, S. H., Clish, C. B. & Gladyshev, V. N. Selenium Deficiency Is Associated with Pro-longevity Mechanisms. *Cell Rep* **27**, 2785-2797 e2783, doi:10.1016/j.celrep.2019.05.001 (2019).

17 Tang, S. *et al.* The exposome in practice: an exploratory panel study of biomarkers of air pollutant exposure in Chinese people aged 60-69 years (China BAPE Study). *Environ Int* **157**, 106866, doi:10.1016/j.envint.2021.106866 (2021).

18 Wang, M. *et al.* Integrated multi-omics uncovers reliable potential biomarkers and adverse effects of zinc deficiency. *Clin Nutr* **40**, 2683-2696, doi:10.1016/j.clnu.2021.03.019 (2021).

19 Matta, K. *et al.* Associations between persistent organic pollutants and endometriosis: A multiblock approach integrating metabolic and cytokine profiling. *Environ Int* **158**, 106926, doi:10.1016/j.envint.2021.106926 (2022).

20 Boughanem, H. *et al.* Linking serum vitamin D levels with gut microbiota after 1-year lifestyle intervention with Mediterranean diet in patients with obesity and metabolic syndrome: a nested cross-sectional and prospective study. *Gut Microbes* **15**, 2249150, doi:10.1080/19490976.2023.2249150 (2023).
